# *p53* Inactivation Upregulates p*73* Expression through *E2F-1* Mediated Transcription

**DOI:** 10.1371/journal.pone.0043564

**Published:** 2012-08-30

**Authors:** Chaitali Tophkhane, Shi-He Yang, Yunbo Jiang, Zhikun Ma, Dharmalingam Subramaniam, Shrikant Anant, Shingo Yogosawa, Toshiyuki Sakai, Wan-Guo Liu, Susan Edgerton, Ann Thor, Xiaohe Yang

**Affiliations:** 1 Department of Pathology, University of Oklahoma Health Sciences Center, Oklahoma City, Oklahoma, United States of America; 2 Department of Molecular & Integrative Physiology, KU Medical Center, The University of Kansas, Kansas City, Kansas, United States of America; 3 Department of Preventive Medicine, Kyoto Prefectural University of Medicine, Kyoto, Japan; 4 Department of Genetics, Louisiana State University Health Sciences Center, New Orleans, Louisiana, United States of America; 5 Department of Pathology, University of Colorado Denver School of Medicine, Aurora, Colorado, United States of America; 6 Julius L. Chambers Biomedical/Biotechnology Research Institute, North Carolina Central University, North Carolina Research Campus, Kannapolis, North Carolina, United States of America; Virginia Commonwealth University, United States of America

## Abstract

While *p73* overexpression has been associated with increased apoptosis in cancer tissues, *p73* overexpressing tumors appear to be of high grade malignancy. Why this putative tumor suppressor is overexpressed in cancer cells and what the function of overexpressed *p73* is in breast cancers are critical questions to be addressed. By investigating the effect of *p53* inactivation on *p73* expression, we found that both protein and mRNA levels of *TAp73* were increased in MCF-7/p53siRNA cells, MCF-7/p53mt135 cells and HCT-116/p53−/− cells, as compared to wild type control, suggesting that *p53* inactivation by various forms upregulates *p73*. We showed that *p53* knockdown induced *p73* was mainly regulated at the transcriptional level. However, although *p53* has a putative binding site in the *TAp73* promoter, deletion of this binding site did not affect *p53* knockdown mediated activation of *TAp73* promoter. Chromatin immuno-precipitation (ChIP) data demonstrated that loss of p53 results in enhanced occupancy of E2F-1 in the TAp73 promoter. The responsive sequence of *p53* inactivation mediated *p73* upregulation was mapped to the proximal promoter region of the *TAp73* gene. To test the role of *E2F-1* in *p53* inactivation mediated regulation of *p73* transcription, we found that *p53* knockdown enhanced *E2F-1* dependent *p73* transcription, and mutations in *E2F-1* binding sites in the *TAp73* promoter abrogated *p53* knockdown mediated activation of *TAp73* promoter. Moreover, we demonstrated that *p21* is a mediator of *p53*-*E2F* crosstalk in the regulation of *p73* transcription. We concluded that *p53* knockdown/inactivation may upregulate *TAp73* expression through *E2F-1* mediated transcriptional regulation. *p53* inactivation mediated upregulation of *p73* suggests an intrinsic rescuing mechanism in response to *p53* mutation/inactivation. These findings support further analysis of the correlation between *p53* status and *p73* expression and its prognostic/predictive significance in human cancers.

## Introduction

Loss of *p53* function, including genetic mutation and functional inactivation, has been associated with carcinogenesis and therapeutic resistance [1], [2]. However, some tumor cells with mutant *p53* remain sensitive to DNA damage. Recent advances suggest that regulation of *p73*, a new *p53* family member, may play a role in this process. Nevertheless, how *p73* is regulated to compensate *p53* function in *p53* inactivated cells remains unclear.

The *p73* gene has two separate sets of promoters that result in the transactivating (TA) and ΔN classes of products known as *TAp73* and *ΔNp73* [3]. Alternative splicing at the carboxyl terminus of both *TAp73* and *ΔNp73* yields further complexity via *p73* isoforms, which include *p73* α, β, γ, δ, ε, ζ, and η [4], [5]. Among them, *p73* α and β are the major TA isoforms. Similar to *p53*, *TAp73s* are able to transactivate some *p53* target genes and induce apoptosis and growth arrest. Functional *p73* has been linked to chemosensitivity [6]. More importantly, *p73* is able to induce apoptosis/growth arrest in cells with mutant *p53* [7]. These findings not only support *p73* as a tumor suppressor but also underscore the significance of *p73* in *p53*-independent anti-tumor mechanisms. In contrast to *TAp73*, *ΔNp73s* have opposing activity and function as oncoproteins by inactivating *p53* and *TAp73* through the formation of heterodimers with these tumor suppressors [3].

Although in vitro studies suggest that *p73* (which indicates *TAp73* if not otherwise specified) is a putative tumor suppressor, the role of *p73* in cancer development remains to be established. Different from *p53*, *p73* mutation in cancer tissues is rare. Interestingly, overexpression of wild type *p73* is frequently detected in different types of human cancers, including breast, lung, prostate, bladder and other types of cancers [8], [9], [10]. In breast cancers, increased *p73* protein and mRNA levels have been detected in approximately one third of the cases that have been studied [11]. While *p73* overexpression has been associated with increased apoptosis in cancer tissues, *p73* overexpressing tumors appear to be of high grade malignancy [12]. Why this putative tumor suppressor is overexpressed in cancer cells and what the function of overexpressed *p73* is in breast cancers are critical questions to be addressed. Among the factors that may contribute to *p73* overexpression, interactions between *p53* and *p73* have been targeted by many investigators. It was reported that increased *p73* mRNA levels was correlated with *p53* mutation detected using mutant *p53* specific antibody (clone 1801) [8]. Other studies using different tissues also suggest that *p73* overexpression/alteration was linked to defective *p53*, as examined by loss of heterozygous (LOH) and immunohistochemistry [13]. However, DNA sequencing of the *p53* gene from 8 cases of breast cancers overexpressing *p73* in a separate report failed to show a similar correlation, which might be due to limited case number and no microdissection of the samples [11]. Therefore, correlation between *p53* status and the expression of individual major isoforms of *p73*, and its clinical relevance in breast cancers require further studies.

Available results suggest that *p53* and *p73* interact in multiple ways. On the one hand, some *p53* mutants, such as R175H and R248W, can bind to *TAp73* and inactivate its function [14]. On the other, overexpression of *ΔNp73* is able to bind to and inactivate *p53* and *TAp73*, thereby functioning as a dominant negative inhibitor [3]. In addition, upregulation of *p53* induces *ΔNp73* transcription and expression, suggesting a feedback network between *p53* and *p73* [15]. It was also reported that overexpression of *p53* or *p73* induced *p73* transcription in certain cell lines [16]. If only based on the above findings, one would conclude that *p53* mutation simultaneously inactivates both *p53* and *p73* systems; and the role of *p73* in *p53* independent apoptosis in *p53* mutant cells would be largely excluded. In fact, *p73* is commonly overexpressed in cancer cells and is functional in many cell lines with mutant *p53* [7]. This indicates that there might be additional mechanisms that coordinate *p53* inactivation and *p73* activation, such that *p73* is upregulated/activated to rescue some, if not all, of *p53* function.

In this study, we investigated the communication between *p53* mutation/inactivation and *p73* expression/activation. Using several *p53* inactivation models we demonstrated that inactivation of *p53* upregulated *TAp73* expression. The underlying mechanisms involve *E2F-1* mediated regulation of *TAp73* transcription modulated by *p21* activity.

## Materials and Methods

### Cell culture and transfection

MCF-7 cells were maintained in DMEM/F12 medium (Sigma, MO) supplemented with 10% fetal bovine serum (FBS). HCT-116 and HCT116p53−/− cells were a gift from Dr. Bert Vogelstein (The Johns Hopkins University). MCF-7 cell line expressing p53SiRNA was established by transfecting MCF-7 cells with p53/SiRNA plasmid (Imagenex, CA), followed by G418 selection. G418 resistant clones were pooled for further characterization. For transient transfection, cells were inoculated 24 hours before transfection. The transfection was performed using FuGENE6 (Roche, IN) according to manufacturer's protocol.

### Plasmid construction

Luciferase reporter plasmids containing full-length *p73* promoter (p73PF, −2713/+77) as well as promoter deletion mutants, *p73NruI* (−1210/+77), *p73SacI* (−883/+77), *p73PstI* (−299/+77) and *p73PvuII* (−220/+77) were constructed as in a previous report [17]. p73pvuII-E2F-1–155/−132 double mutant was generated in a previous study [18]. To construct the luciferase reporter vector containing full length *p73* promoter that lacks *p53*-binding site, the 61-bp fragment containing putative *p53* binding sequence was eliminated by use of following primers: Δ61F: 5′ GCC ATG AAG ATG TGC GAG T 3′ and Δ61R: 5′ GAA GTT CAT GGC CGC CGC CTG CCG C 3′. The resulting PCR product was cloned into pGVB2. This construct lacking *p53* binding site was designated as p73-PFΔ61. pcDNA3-p21 plasmid was a gift of Dr. Todd Sladek.

### Electrophoretic Mobility Shift Assay

Sixty-one oligonucleotides corresponding to wild type and mutant *p53*-binding sequences were synthesized and radiolabeled with [γ-^32^P] dATP by using T4 polynucleotide kinase. DNA binding reactions were performed in 20 µl containing 10 µg of protein from nuclear lysates of MCF-7, MCF-7/SiRNA, HCT116 or HCT116/p53^−/−^ cells, 4 µl of 5× binding buffer (50 mM Tris pH 8.0, 750 mM KCl, 2.5 mM EDTA, 0.5% Triton-X 100, 62.5% glycerol (v/v), 1 mM DTT), 2 µl of polydIdC, and 50000 cpm of radiolabeled probe per sample. Twenty nanograms of unlabeled probes corresponding to wild type or mutant oligonucleotides were used for competition. Antibody supershift was performed using 3 µg of anti-p53 antibody. Binding reactions were incubated at room temperature for 30 minutes and then resolved on 4% PAGE at room temperature (5–6 hours at 70v). Gels were dried and exposed to film at −80°C.

### Luciferase Reporter Assays

Cells were cultured at 1×10^5^ cells/well in 12-well tissue culture plates 24 hours prior to transfection. The reporter plasmid was cotransfected with pSV-β-gal and appropriate plasmids, such as pcDNA3, pcDNA3/p53, pcDNA3/E2F-1 or pcDNA3/p21, using FuGENE 6 by following the standard protocol. Cells were harvested 40 hours post transfection and luciferase activity was measured using Luciferase Reporter Assay System (Roche, MN) according to the manufacturer's instructions on a luminometer. Results were normalized with β-gal activity and presented as fold increase over control luciferase activity. All experiments were performed in triplicates.

### Western Blotting

Fifty µg of protein lysate was loaded onto each lane of a gel. Proteins were separated with 12% SDS-PAGE and transferred to PVDF membrane. The membrane was probed with a specific primary antibody at appropriate dilutions, followed by washing and probing with a corresponding secondary antibody. The specific protein band was visualized by autoradiography using an ECL kit (GE Health Care). Antibodies against *p53*, *E2F-1*, *p21* and Actin were purchased from Santa Cruz Biotechnology (Santa Cruz, CA). Antibody against *p73* (AB3) was purchased from Calbiochem.

### RNA Extraction and RT-PCR

Total RNA was isolated from control and treated cells using RNeasy Mini Kit (QIAGEN, CA). First strand cDNA synthesis was performed using SuperscriptIII™ First Strand synthesis system (Invitrogen). The primer sequences used were as follows: TAp73 forward (FW): 5′-GCA CCA CGT TTG AGC ACC TCT-3′; TAp73 reverse (RV): 5′-GCA GAT TGA ACT GGG CCA TGA-3′; DNp73 FW: 5′-CAA ACG GCC CGC ATG TTC CC-3′; DNp73 RV: 5′-TTG AAC TGG GCC GTG GCG AG-3′; Actin FW: 5′-GCA CCA CAC CTT CTA CAA TGA GC-3′ Actin RV: 5′-GAC GTA GCA CAG CTT CTC CTT AAT G-3′; PCR reactions were performed in a 50 µl reaction volume for 30–35 cycles. Twenty microliters of PCR product was electrophoresed on 1.8% agarose gel and visualized by Ethidium Bromide staining. Quantitative detection of TA and DNp73 mRNA levels was performed with real time RT-PCR using a Bio-Rad CFX96 Touch™ Real-Time PCR Detection System.

### ChIP Assays

ChIP assays were carried out with a ChIP assay kit from the Upstate Biotechnology, Inc. (NY). MCF-7/control and MCF-7/sip53 cells were grown on 100 mm plates to 90% confluence in DMEM/F12 medium containing 10% FBS before collection. The cells were cross-linked with 1% formaldehyde (final concentration) by direct adding to the cell medium for 10 minutes at 37°C, followed by washing with cold PBS containing 1× protease inhibitor cocktail. The cells were then scraped, centrifuged for 5 minutes at 4°C, 4000 rpm, and lysed by incubation with 1 mL of l× SDS lysis buffer for 10 minutes on ice. The lysates were subjected to 15 cycles of sonication (15 sec pulse/2 min incubation for each cycle) at 50% duty cycle using an Out Control intensity of 2 (VirSonic 475) followed by centrifugation at 4°C, 16000 rpm for 10 minutes. Three hundred micrograms of supernatant protein were diluted 10-fold with ChIP dilution buffer and immunocleared with 70 µl of Protein A Agarose/Salmon Sperm DNA (50% Slurry) for 1 hour at 4°C with agitation. Collected agarose beads were saved as control IgG. Immunoprecipitation was performed overnight at 4°C with E2F-1 antibody (SC-20, Santa Cruz Biotechnology Inc.) at 3 µg/mL of precleared supernatant. Immunocomplexes were extracted by adding 65 µl of Protein A Agarose/Salmon Sperm DNA (50% Slurry) for 1.5 hour at 4°C followed by gentle centrifugation (1000 rpm, 1 minute, 4°C). Precipitates were washed sequentially with 1 mL of low-salt washing buffer, high-salt washing buffer, LiCl washing buffer, and were washed twice with 1 mL of TE buffer and extracted twice with 250 µl of freshly made elution buffer (1% SDS, 0.1 M NaHCO3). The pooled eluates in a total volume of 500 µl were mixed with 20 µL of 5 M NaCl, heated at 65°C for 10 hours to reverse the formaldehyde crosslinking. DNA fragments were then purified by phenol/chloroform and precipited with ethanol.

Five µL from the 30 µL DNA extraction was amplified by PCR with the following pairs of primers for the proximal promoter region of the TAp73 gene: Forward: 5′-GCCCATATAACCCGCCTA-3′; and Reverse: 5′-CCCTGGGCCTCCTACCTG-3′. The PCR conditions were: initial 10 minutes of denaturing at 94°C followed by 35 cycles of denaturing for 30 seconds at 94°C, annealing for 30 seconds at 59°C, and elongating for 45 seconds at 72°C; the final extension took place at 72°C for 5 minutes. Equal volumes of each PCR sample were subjected to electrophoresis in a 2% agarose gel, followed by gel documentation.

## Results

### *p53* knockdown/mutation induces *p73* upregulation in cancer cells

**Figure 1:**
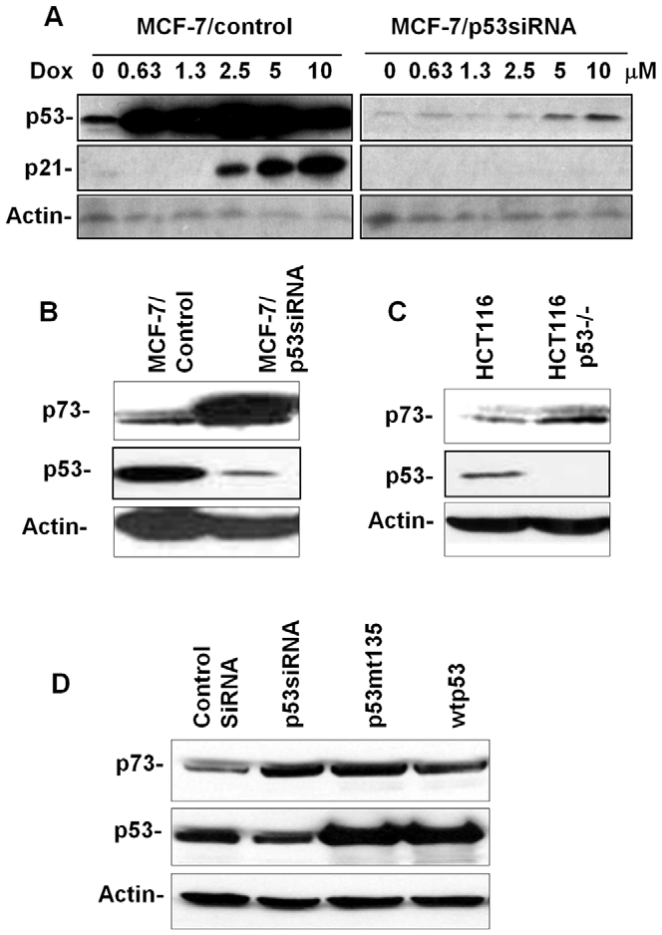
A. *p53* knockdown in MCF-7 cells. A stable MCF-7 subline was established by transfection pGeneSupressor/p53siRNA followed by G418 selection. Control and MCF-7/p53siRNA cells were treated with doxorubicin (Dox) at indicated concentrations for 20 h. Protein levels of *p53*, *p21* and actin were detected by Western blot. B & C. Upregulation of *p73* β in MCF-7 (B) and HCT-116 (C) cells with *p53* knockdown/knockout. Protein levels of *p53*, *p73* β and Actin in control and *p53* knockdown/knockout cells were detected with Western blot. D. Regulation of *p73* by *p53* in transient transfection system. MCF-7 cells were transiently transfected with control vector, pGeneSupressor/p53siRNA, pCMV/mtp53 (135G/A) and pCMV/wtp53, respectively. Protein levels of *p53*, *p73* β and Actin were detected using Western blot.

To investigate the specific role of *p53* in the regulation of *p73*, we established a stable MCF-7 subline in which wild type *p53* was knocked down using *p53*-targeting siRNA (MCF-7/p53siRNA). As shown in Fig. 1A, *p53* knockdown was highly efficient, as indicated by the striking difference in *p53* and *p21* levels between control and MCF-7/p53siRNA cells in response to DNA damaging agent doxorubicin. We next examined *p73* expression in the paired cell lines and found that *p73* protein levels were significantly increased in MCF-7/p53siRNA cells (Fig. 1B), suggesting that loss of *p53* may upregulate *p73* expression. This hypothesis was supported by the increase of *p73* levels in HCT116/p53−/− cells, as compared to the wild type control (Fig. 1C). To exclude the possibility of clonal selection in the stable cell lines and to demonstrate that *p53* mutation may also induce *p73* upregulation, MCF-7 cells were transiently transfected with control vector, p53siRNA, mutant *p53* (mtp53) (G135A) and wild type *p53* (wtp53), followed by *p73* detection. We found that transfection of p53siRNA resulted in decreased *p53* but increased *p73* levels (Fig. 1D), which was consistent with the data from the stable cell lines. In the cells transfected with mtp53 (G135A), protein levels of both *p53* and *p73* were increased. Because dimer formation between mutant and wild type *p53* increases *p53* stability and inactivates wtp53 [19], increased *p73* levels in these cells suggest that *p53* inactivation by point mutation may also induce *p73* expression. Interestingly, p73 protein levels in the cells transfected with wtp53 were modestly increased, instead of decrease. This may suggest the complexity of p53–p73 interaction, which will be discussed later. Taken together, these data demonstrated that *p53* inactivation, either by knockdown/knockout or by mutation, upregulates *p73* protein levels in these cells.

### *p53* inactivation mediated upregulation of *p73* is primarily regulated at the transcriptional levels

**Figure 2:**
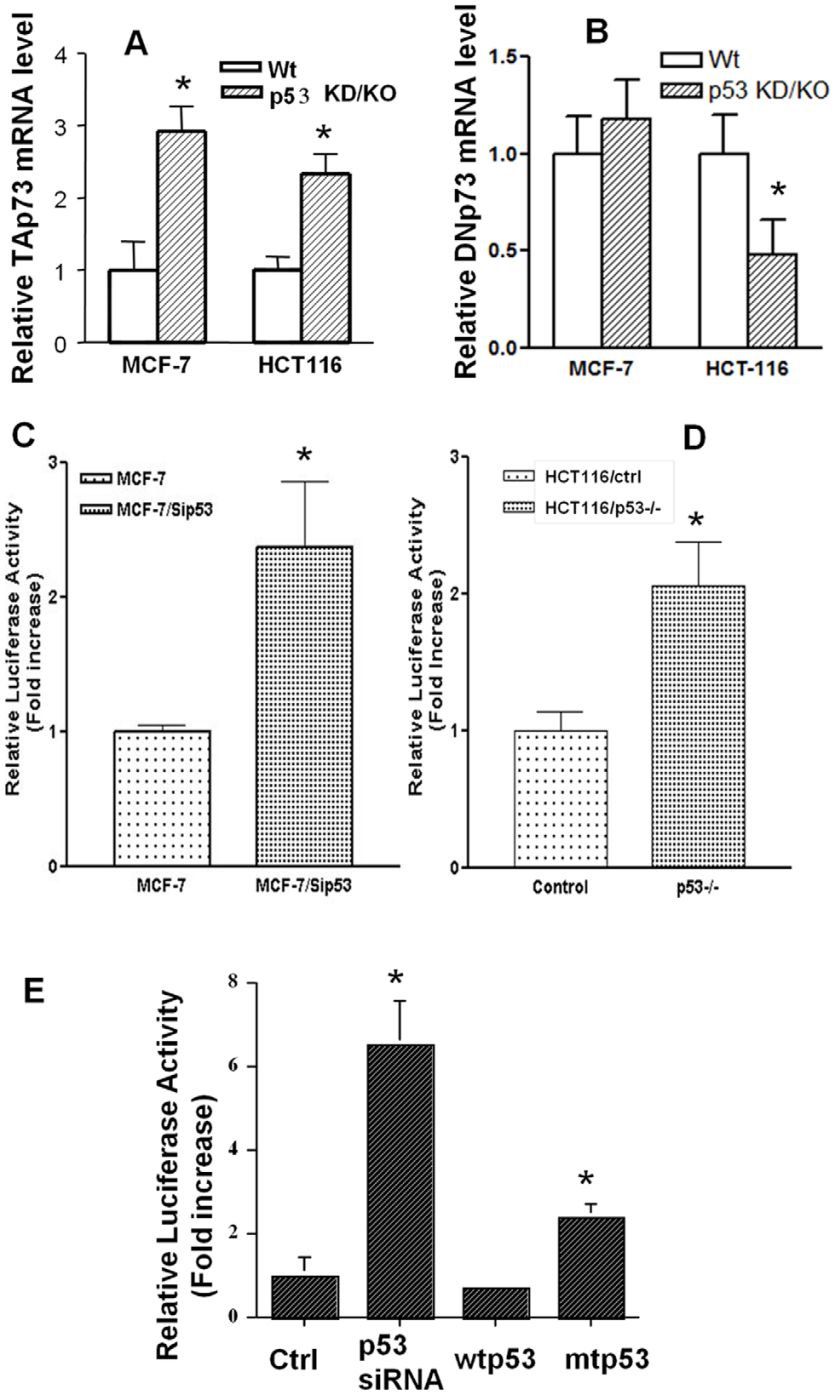
*p53* inactivation mediated upregulation of *p73* is primarily regulated at the transcriptional level. A & B. p53 inactivation upregulates TAp73 but not DNp73 mRNA levels. Relative mRNA levels of *TAp73* (A) and DNp73 (B) in control cells (with wtp53) and *p53* knockdown/knockout (p53KD/KO) MCF-7 and HCT-116 cells were detected with real-time RT-PCR using *TAp73* and DNp73 specific primers. Specific mRNA levels were normalized with β-Actin mRNA. C & D. Loss of *p53* induces the activation of *p73* promoter in stable MCF-7 and HCT-116 sublines. Cells of each subline were co-transfected with luciferase reporter construct encoding full length (−2713 to +77) *p73* promoter (p73-PF), pSV-β-gal. Luciferase activity was measured 40 hours after transfection. The experiments were performed at least three times in triplicates. E. Transfaction of p53 siRNA and mutant p53 induces *p73* promoter activity in MCF-7 cells: MCF-7 cells were co-transfected with reporter construct encoding full length *p73* promoter (p73-PF), pSV-β-gal, and vectors of control siRNA, p53siRNA, wild type *p53* (wtp53) or mutant *p53* (mtp53). Luciferase activity was determined as described above. (* p<0.01).

To test whether *p53* inactivation mediated *p73* upregulation occurs at transcriptional and/or translational levels, we examined the mRNA levels and protein half-life of TAp73 in cells with different *p53* statuses. Using cycloheximide blockage assay we found that TAp73 protein degradation was similar between MCF-7/control and MCF-7/p53siRNA cells (data not shown). In contrast, RT-PCR amplification of *p73* mRNA from both MCF-7 and HCT116 paired cell lines indicated that *TAp73* mRNA levels were significantly upregulated in the cells with *p53* inactivation (Fig. 2A).

It was previously shown that p53 induces the expression of its antagonist DNp73 in H1299, Saos2 and LS174T cells, which forms an autoregulatory feedback loop [15]. Therefore, we also examined the transcription of DNp73 in these two paired cell lines. As shown in Fig. 2B, DNp73 mRNA levels were insignificantly increased in MCF-7/p53siRNA cells but remarkably decreased in HCT-116/p53−/− cells, as compared to respective controls. These data suggest that the impact p53 inactivation on *TAp73* and *DNp73* transcription was different. We focused on the regulation of TAp73 in this study.

We next characterized *p53* inactivation mediated regulation of TA*p73* transcription using reporter gene assay. The reporter construct used was the luciferase gene driven by a promoter fragment of the *TAp73* gene spanning from −2713 to +77 of the starting codon (p73PF) [17]. Results from two paired cell lines, MCF-7 control vs. MCF-7-p53siRNA and HCT116 control vs. HCT116p53−/−, showed that p53 knockdown/knockout in both cell lines strongly activated the *TAp73* promoter (Fig. 2 C &D), which supported the transcriptional regulation of *p73* by *p53* inactivation. By testing the activation of the *TAp73* promoter in a transient transfection system, we found *TAp73* promoter activity was increased in MCF-7 cells transfected with p53siRNA or mtp53 (G135A), and it was decreased in response to wtp53 transfection (Fig. 2E). Taken together, these data indicate that *p53* inactivation mediated upregulation of *p73* is primarily regulated at the transcriptional level.

### *p53* inactivation mediated upregulation of *p73* is independent of *p53* binding to the *TAp73* promoter

**Figure 3:**
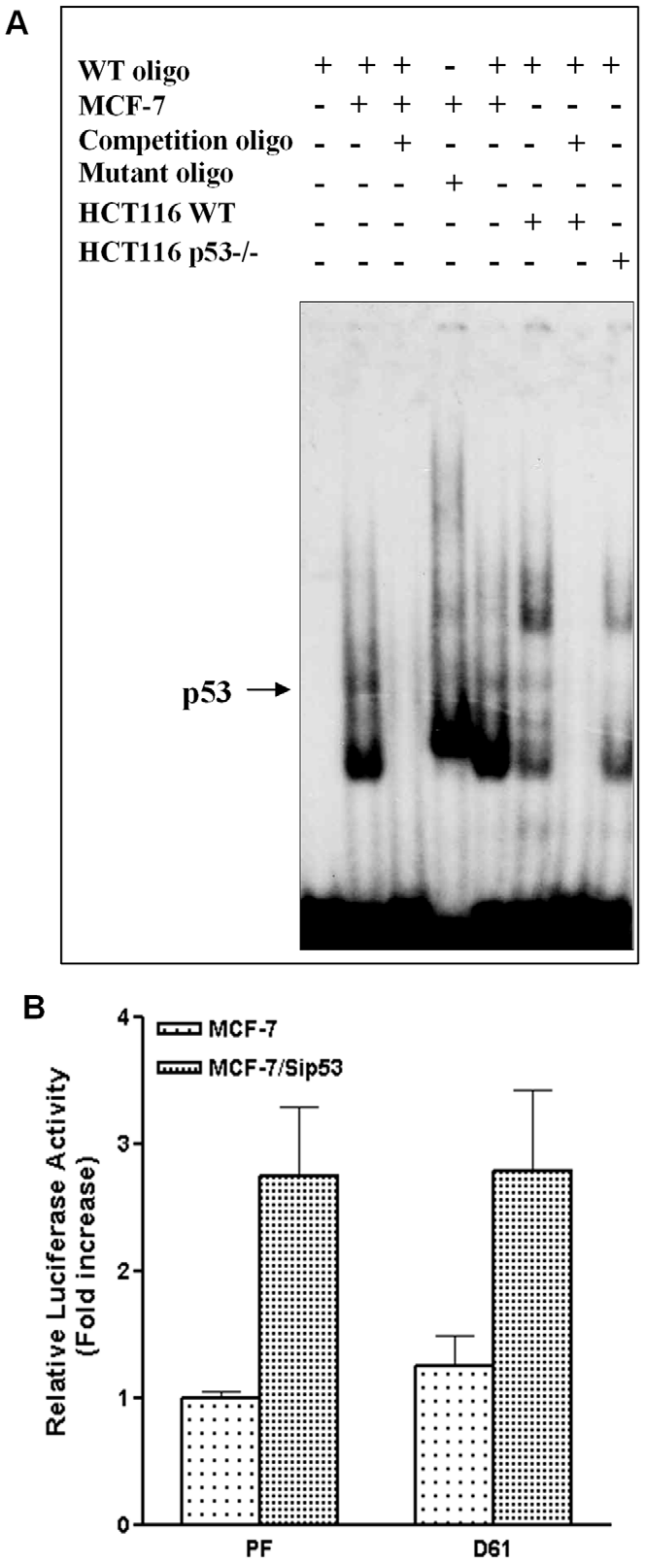
*p53* inactivation mediated upregulation of *p73* is independent of *p53*'s binding to the *p73* promoter. A. *p53* binds to the *p73* promoter. EMSA was performed using 32P-labeled 61 bp oligonucleotide (−2634 to −2574) containing putative *p53*-binding sequence in the *p73* promoter and nuclear extracts of MCF-7 control and MCF-7/p53siRNA cells. Control and *p53* knockout (p53−/−) HCT116 cells were used as control. Arrow indicates *p53* bound to the *p73* promoter sequence. B. Deletion of *p53* binding site in the *p73* promoter did not abrogate *p53*-knockdown mediated upregulation of *p73* promoter activity. MCF-7 cells were transfected with the luciferase reporter constructs encoding full length *p73* promoter, p73PF (PF) or the promoter lacking 61 bp *p53* binding sequence, p73-PFΔ61 (D61) in the presence or absence of *p53* knockdown with siRNA.

Since *p53* is a transcription factor, it is necessary to address whether *p53* inactivation mediated upregulation of *TAp73* is DNA binding dependent. Previously, it was reported that the *TAp73* promoter contains a putative *p53* responsive element (p53RE) between −2634 and −2574. Reporter assays based on a chimeric promoter containing the p53RE suggested that it may function as an enhancer [16]. However, reports from later studies showed that co-transfection of *p53* did not activate the *TAp73* promoter [6], [15], [20]. To determine whether this p53RE plays any roles in p53 inactivation modulated *p73* regulation, we examined DNA binding activity of *p53* to this putative binding site using electrophoretic mobility shift assay (EMSA). The labeled probe, which was a 61 bp oligonucleotide fragment (−2634 to −2574) containing the putative binding site, was incubated with nuclear protein lysate extracted from MCF-7, HCT116 or HCT116p53−/− cells in the presence/absence of competitive oligonucleotides. As shown in Fig. 3A, the probe formed a weak complex with the nuclear protein extract from both wild type MCF-7 and wild type HCT116 cells, but not with the p53−/− extract nor in the presence of competitive wild type oligomers. This signifies that *p53* was able to bind to the putative binding site. To determine whether this DNA binding activity plays a role in *p53* inactivation mediated upregulation of *p73*, we deleted this putative *p53* binding sequence in the full-length *p73* promoter (p73PF) to create a reporter construct named p73PFΔ61. By transfecting p73PF or p73PFΔ61 into control and p53siRNA expressing MCF-7 cells, we found that deletion of putative *p53* binding sequence from the full-length *p73* promoter did not affect *p53* knockdown-mediated increase in the *p73* promoter activity (Fig. 3B). These results suggest that *p53* binding site in the *p73* promoter is not involved in *p53* knockdown-mediated upregulation of *p73* transcription, although *p53* has some affinity to this site.

### The responsive sequence of *p53* inactivation mediated transcriptional activation of *p73* is mapped to the proximal promoter region of the *TAp73* promoter

**Figure 4:**
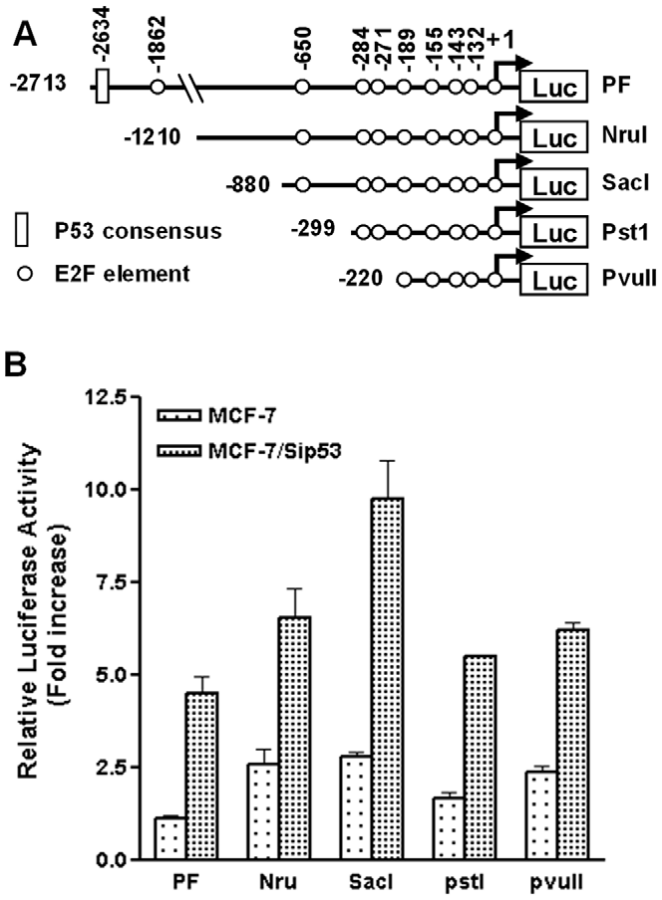
Mapping of the DNA sequence that is responsible for *p53* inactivation mediated *p73* upregulation. A. Reporter constructs of *TAp73* promoter with a serious of deletions from the 5′ end. Putative binding sites for *E2F* and *p53* are depicted. The drawing is not proportional to the actual size. B. MCF-7 cells were co-transfected with luciferase reporter constructs with different lengths of *p73* promoter, pSV-β-Gal, and vectors of control siRNA or p53siRNA. Luciferase activity was measured 40 hours after transfection, which was followed by β-galactosidase normalization.

To identify the minimal promoter region that is required for *p53* inactivation-mediated enhancement of *p73* transcription, we transfected a series of reporter constructs with various deletions in the *TAp73* promoter region (Fig. 4A) into MCF-7/control and MCF-7/p53siRNA cells, respectively. Luciferase analysis revealed that *p53* inactivation was able to enhance the promoter activity of each construct (Fig. 4B). The response of the shortest fragment (p73-pvuII, −220 to +71) was even greater than that of the full length promoter (p73PF). These results suggest that this short fragment upstream of the start codon was sufficient for *p53*-knockdown-mediated upregulation of *p73* transcription.

### *p53* inactivation upregulates *p73* transcription through *E2F-1*

**Figure 5:**
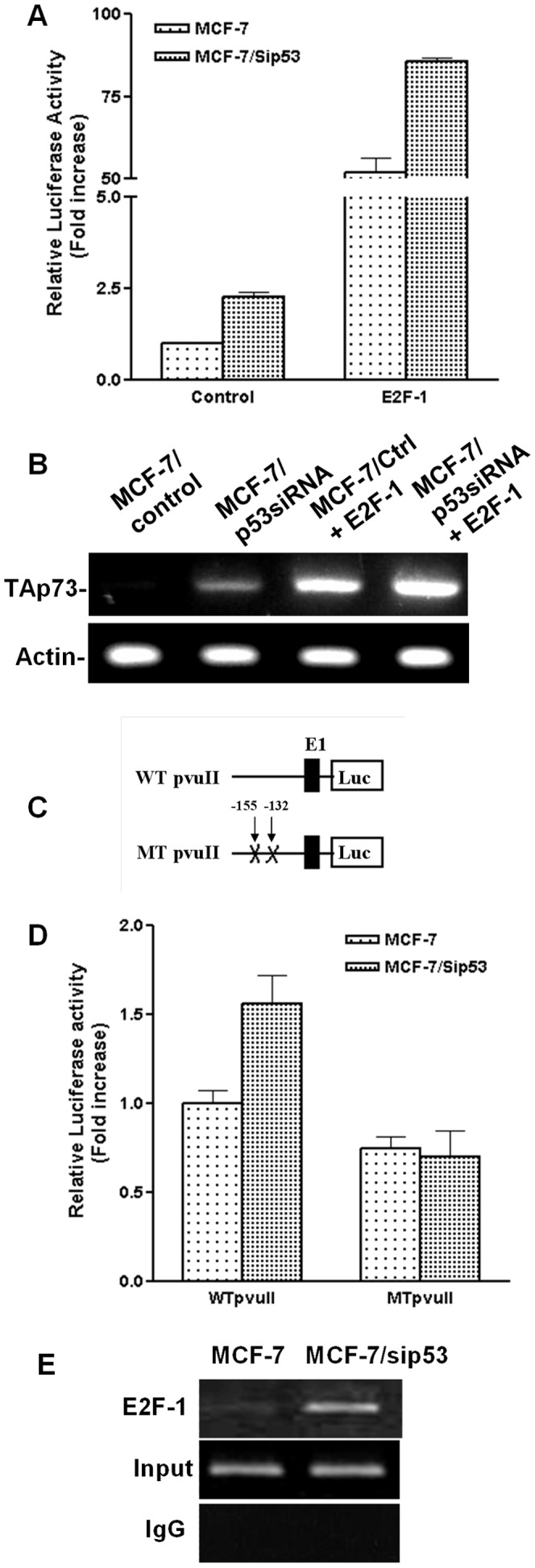
Critical role *E2F-1* in *p53* knockdown-mediated upregulation of *p73* transcription. A. Additive effect of *E2F-1* overexpression on *p53* knockdown mediated increase in *p73* promoter activity. MCF-7 cells were co-transfected with p73-PF/luciferase, pSV-β-Gal, and control or p53siRNA plasmids either in presence or in absence of *E2F-1*. Luciferase and β-galactosidase activity was determined 40 hours post-transfection. B. *TAp73* mRNA levels in MCF-7 cells transfected with control vector, p53siRNA and/or *E2F-1* cDNA. MCF-7 cells were transiently transfected with either control siRNA or *p53*-specific siRNA either in the presence or in the absence of *E2F-1*. RT-PCR analysis was performed using gene-specific primers. C & D. *E2F-1* binding is required for *p53* knockdown mediated increase in *p73* promoter activity. MCF-7 cells were co-transfected with control or p53siRNA vector and reporter construct encoding wild type or mutant *p73* promoter (p73PVUII, −220 to +71), in addition to pSV-β-Gal plasmid. The mutant PVUII promoter fragment contains mutant *E2F-1* binding sites at −155 and −132 (C). Luciferase and β-gal activity was determined 40 hours post- transfection (D). E. Occupancy of the E2F responsive element in the TAp73 promoter by E2F-1 is enhanced in MCF-7/p53siRNA cells. Detected with chromatin immunoprecipitation (ChIP) assay, DNA fragment of the TAp73 promoter was amplified from the complexes immunoprecipitated with E2F-1 antibody from the paired cell lines. Input row were the DNA fragment amplified from the extracts before immunoprecipitation. In the control immunoglobulin G (IgG) reaction, PCR was done in the eluates from beads collected after preclearing of these extracts with normal rabbit serum.

Since we had not found any potential *p53* binding site in the first 220 bp region of the *TAp73* promoter, we speculated that *p53* inactivation might regulate *p73* transcription through indirect mechanisms. Because *E2F-1* is a major transcription factor that regulates *TAp73* transcription and there are multiple *E2F* binding sites in the proximal promoter region [18], [21], [22], we reasoned that modulation of *E2F-1* activity might play a role in *p53* inactivation mediated upregulation of *p73* transcription. To test our hypothesis, we co-transfected p73PF reporter gene with pcDNA3/E2F-1 or control vector into MCF-7/control and MCF-7/p53siRNA cells, respectively. We found that overexpression of *E2F-1* drastically enhanced the activation of the *TAp73* promoter, especially in MCF-7/p53siRNA cells (Fig. 5A). Consistent with the reporter assays, results from RT-PCR detection of *TAp73* mRNA in the cells with the same transfection settings also showed that transcription of *TAp73* was significantly boosted in MCF-7/p53siRNA cells (Fig. 5B).

To further demonstrate the specific role of *E2F-1* in *p53* inactivation regulated *p73* transcription, we tested whether mutation of *E2F* binding sites abolishes *p53* inactivation mediated upregulation of *p73* transcription. It has been shown that, although there are multiple putative *E2F* binding sites in the proximal region of the *TAp73* promoter, simultaneous mutation of two major *E2F* binding sites, −132 and −155, reduces the promoter activity by more than 90% [18]. We therefore transfected reporter constructs with wild type p73pvUII (wtp73pvuII) or p73pvUII with double mutations at −132/−155 (mtp73pvuII) (Fig. 5C) into MCF-7/control and MCF-7/p53siRNA cells, respectively. As shown in Fig. 5D, in contrast to induction in the cells transfected with wtp73pvuII, there was no upregulation of luciferase activity in MCF-7/p53siRNA cells transfected with mutant reporter construct (mtp73pvuII).

To support the reporter assay data, we performed chromatin immunoprecipitation (ChIP) assay to determine the effect of p53 inactivation on E2F-1 binding to the p73 promoter. As shown in Fig. 5E, PCR reaction targeting the p73 promoter region amplified a greater amount of DNA from the E2F-1 immunoprecipitates derived from MCF-7/p53siRNA cells, indicating that loss of p53 resulted in enhanced occupancy of E2F-1 in the TAp73 promoter (Fig. 5E). These results demonstrate that modulation of *E2F* activity plays a critical role in *p53* inactivation mediated upregulation of *p73* transcription.

### *p21* is a mediator of *p53-E2F* crosstalk in the regulation of *p73* transcription

**Figure 6:**
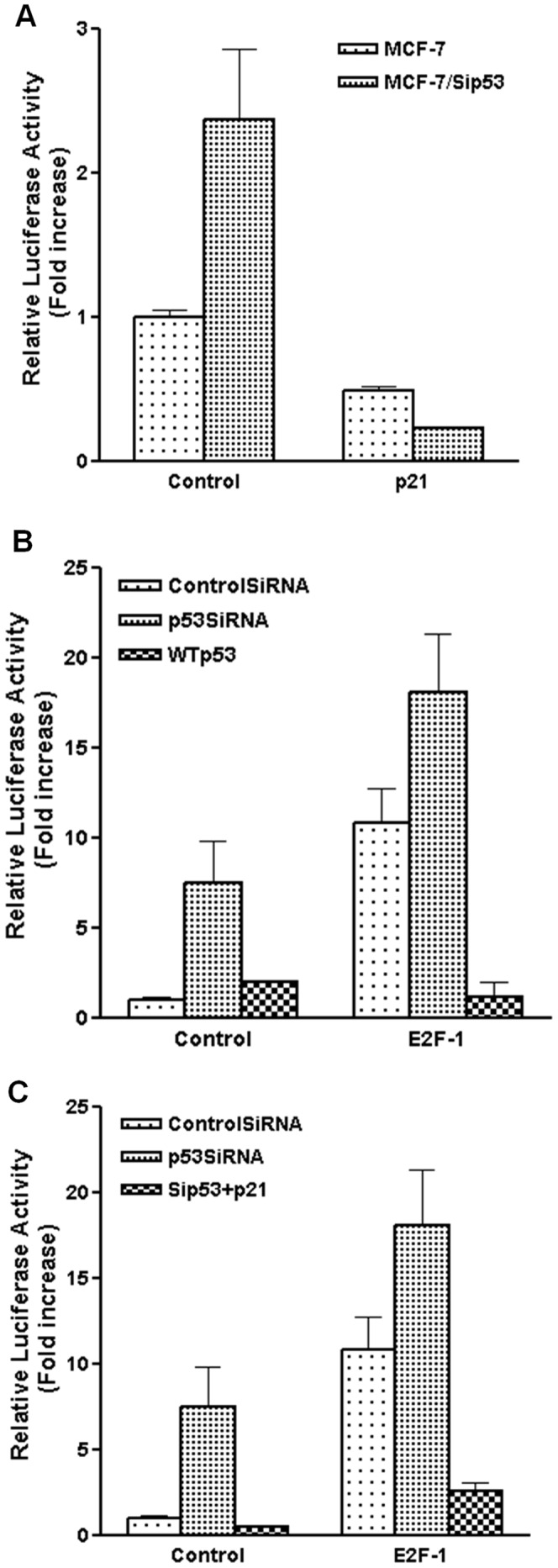
*p21* is a mediator of *p53* inactivation induced upregulation of *p73* transcription. A. Overexpression of *p21* abolishes *p73* upregulation in control and MCF-7/p53siRNA cells. MCF-7/control and MCF-7/p53siRNA cells were cotransfected with p73-PF/pSV-β-Gal and control vector or pcDNA3/p21. Cell lysate was collected for luciferase assay 40 hours post-transfection. All the experiments were performed at least three times in triplicates. B. Overexpression of wtp53 reverses p53siRNA induced *p73* transcription in the presence or absence of *E2F-1* overexpression. p73-PF/pSV-β-Gal and pcDNA3/E2F-1 or control vector were cotransfected with the plasmids encoding control siRNA, p53siRNA or wtp53 into MCF-7 cells. Luciferase activity was determined as described above. C. Overexpression of *p21* abrogates p53siRNA induced *p73* transcription in the presence or absence of *E2F-1* overexpression. p73-PF/pSV-β-Gal and pcDNA3/E2F-1 or control vector were cotransfected with the plasmids encoding control siRNA, p53siRNA or pcDNA3/p21 into MCF-7 cells. Luciferase activity was determined as described above.

To bridge the gap between *p53* and *E2F-1* interactions in the regulation of *p73* transcription, we tested the role of *p21* in this crosstalk. *p21* is a cyclin-dependent kinase inhibitor and a major transcriptional target of *p53* [23]. It is known that *p21* blocks retinoblastoma protein (pRb) phosphorylation by inhibition of cyclin/CDK complexes [24], [25], which constrains the transcriptional activity of *E2F*. To determine the role of *p21* in *p53* inactivation mediated upregulation of *p73*, we co-transfected p73PF reporter gene with pcDNA3/p21 or control vector into MCF-7/control and MCF-7/p53siRNA cells, respectively. As shown in Fig. 6A, transfection of *p21* cDNA reversed the increase of *p73* promoter activity by p53siRNA. Consistently, in contrast to p53siRNA induced upregulation, overexpression of wtp53 inhibited *p73* promoter activation, especially in the presence of *E2F-1* overexpression (Fig. 6B). We further demonstrated that overexpression of *p21* abolished p53siRNA induced activation of *TAp73* promoter in the absence or presence of *E2F-1* overexpression, in a pattern similar to wtp53 overexpression (Fig. 6C). Taken together, these data suggest that downregulation of *p21* and the consequent activation of *E2F-1* activity are critical mediators of *p53* knockdown-induced upregulation of *p73* transcription.

## Discussion

As a *p53* family member and a critical molecule involved in apoptosis, cell cycle regulation and differentiation, *p73* is regulated at multiple levels. It is known that *p73* expression is regulated by multiple splicing, alternative promoters, and alternative initiation of translation [26], [27]. Methylation of *p73* promoter has also been detected in human tumors [28], [29]. In response to cellular stresses, such as DNA damage, *p73* transcription and activity can be regulated by phosphorylation, acetylation, and interacting with other cellular factors [30], [31], [32]. In particular, the interaction between *p53* and *p73* has been a critical issue in the understanding of the role of *p73* in tumor development and therapeutic responses [33], [34]. Our data, which demonstrate *p53* inactivation mediated upregulation of *TAp73* in cancer cells, reveal a novel mechanism that regulates *p53*–*p73* interactions.

Overexpression of wtp73 has been frequently detected in human cancers [8], [9], [10]. Immunohistochemical (IHC) data suggest a correlation between *p73* overexpression and *p53* mutation [8], [13]. Although it was difficult to discern suppressor (*TAp73*) from oncogenic (*DNp73*) isoforms in IHC tests, examination of specific isoforms of *p73* mRNA indicated that both suppressor and oncogenic *p73* were significantly upregulated in tumor tissues [35]. Our preliminary data also showed a correlation between p53 (mAb1801) staining, a surrogate marker of p53 mutation and p73 expression (data not shown). However, while overexpression of *DNp73* was linked to wtp53 activation [35], the mechanism of *TAp73* overexpression in cancer tissues remains poorly understood. We approached this critical issue by examination of *p73* expression in paired cell lines with specific knockdown/knockout of wtp53. We found that inactivation of *p53* in MCF-7 and HCT-116 cells resulted in increased expression of *TAp73*, but not *DNp73*, which suggests that *p53* inactivation-mediated-upregulation of *p73* may contribute to the *p73* overexpression detected in human cancers. In addition to *p53* knockdown and knockout, we found that inactivation of *p53* by overexpression of mtp53 (G135A) led to *p73* upregulation, indicating that *p53* inactivation by means other than deletion/knockdown also induce *p73* expression. However, because there are numerous *p53* mutations and these mutations may have various functional outcomes [36], [37], the effect of specific *p53* mutations on *p73* transcription requires further analysis. Nevertheless, our data support a correlation between loss of *p53* function and increase of *p73* transcription.

We showed that *p53* inactivation mediated upregulation of *p73* was primarily regulated at the transcriptional level, and this activity was mapped to the proximal region of the *p73* promoter. Previously, it was reported that there was a potential *p53* binding site at the distal region (∼−2634 to−2574) of *p73* promoter and expression of wtp53 in SAOS cells induced *p73* expression [16]. In our tests, we found that the radio-labeled probe that contained the potential *p53* binding site was able to form a weak complex with *p53* from wild type MCF-7 and HCT-116 cells. However, results from reporter gene assays with series deletion of *p73* promoter indicated that *p53* inactivation was able to activate the luciferase reporter construct that only contained the proximal region *p73* promoter (−220 to +71). These data suggest that *p53* inactivation upregulated *p73* transcription, which was independent of *p53* binding. Increased activation of the full length *p73* promoter (p73PF), which includes the *p53* binding site, by *p53* inactivation suggest that, at least in the cell lines tested, DNA binding independent regulation overrides DNA binding effect in *p53*–*p73* communication.

Based on our evidence showing that p53 inactivation upregulates p73 transcription, one would expect a decrease of p73 expression in the cells transfected with wtp53. However, p73 protein levels in these cells were not decreased or even modestly increased (Fig. 1D). This might be due to the involvement of other mechanisms in the case of wtp53 overexpression, which are not symmetrical to p53 knockdown or knockout. Consistently, previous reports by different authors have shown that the TAp73 promoter is not modulated by p53 overexpression in co-transfection experiments [6], [15], [20]. In context with the transcriptional data in Fig. 2E, which shows no increase in TAp73 promoter activation upon wtp53 transfection, the results suggest that increased p73 protein levels in the presence of p53 overexpression or transfection-associated stress might induce modified protein stability and/or additional mechanisms. Given the complex nature of *p53*–*p73* communication, differentiation of these mechanisms in *p53*–*p73* crosstalk requires further investigation.

Our data further demonstrate that modulation of *E2F-1* regulated transcription of *p73* is responsible for upregulated *p73* by *p53* inactivation. It is known that *E2F* is a powerful transcription factor that regulates a number of molecules involved in cell cycle progression and apoptosis, including *TAp73* [38]. *E2F* activity is tightly regulated by phosphorylation status of pRb, a well known tumor suppressor [38]. Hence, our results not only address how *p53* inactivation upregulates *p73* transcription in a DNA binding independent manner but also underscores the role of pRb/E2F-1 pathway in the regulation of *p73* expression.

Based on previous reports, *p53*-*E2F* interaction may regulate *E2F* target genes in different ways. For the regulation of cdc2, an *E2F* target gene, it was reported that *p53* represses the cdc2 promoter by inducing *p21*. Increased *p21* inhibits cyclin-dependent kinase activity that enhances the binding of *p130* and *E2F4*, which together bind to and repress the cdc2 promoter [39]. It was also reported that *p53* represses survivin expression by interfering with *E2F* mediated transcription through forming E2F-1/p53 complex in a DNA binding dependent manner [40]. Because we found no p53 binding site in the p73PVUII fragment, and mutation of two major *E2F* sites or overexpression of *p21* was sufficient to abolish *p53* mediated upregulation of *p73*, it is likely that decreased *p21* in *p53* inactivated cells may enhance *E2F* mediated transcription of *p73*. However, because *p53* may also affect *E2F* activity through other factors, such as *p300* [41], it is possible that *p53* inactivation modulates *E2F* mediated *p73* transcription by additional mechanisms. This may partially explain our observation that *p53* knockdown/knockout mediated upregulation of *p73* is more evident than *p53* overexpression mediated inhibition of *p73*.

Association between p53 and p73 status and its impact on clinical outcomes are complex issues. This is complicated by the existence of functionally opposite p73 isoforms and redundant alterations. In context with previous reports, it appears that *p53* may communicate with *p73* by multiple means. These include dimers between certain *p53* mutants and *p73* that downregulates *p73* activity [33], [42]; a potential interaction between *p53* and the *p73* promoter at the distal region [16]; p53 mediated induction of oncogenic DNp73; and our finding that *p53* inactivation upregulates *p73* transcription. Different from the others, *p53* inactivation mediated upregulation of TA*p73* transcription could be a mechanism that explains the compensatory role of *p73* in *p53* mutant or inactivated cells. However, how this regulation contributes to the regulation of p53 independent apoptosis and tumor suppression requires further investigation. The outcomes would be determined by the balance between oncogenic and tumor suppressor p73 isoforms. In this context, our data showed that p53 inactivation induced remarkable decrease of DNp73 in HCT-116 cells but not MCF-7 cells, which is consistent with previous report that p53 activation induces expression of DNp73 in some cancer cells. Nevertheless, since p53 and E2F status might be also associated with the expression of other isoforms, such as ΔEx2/3p73, a comprehensive analysis of p53 inactivation regulation of other p73 isoforms/variants will be followed.

Taken together, our results reveal that *p53* inactivation may upregulate TA*p73* expression through *E2F-1* mediated transcriptional regulation, which involves modulated *p21* activity. These findings support further molecular analysis of the correlation between *p53* status and TA*p73* expression in human cancers, as suggested by the IHC data [8], [13]. Studies on the functional impact of p73 upregulation on apoptosis and tumor suppression in cancer cells lacking wtp53 will advance our understanding of this complex issue. Moreover, given the role of *E2F-1* in the regulation of *p73* transcription and *p53*–*p73* communication, cancer therapeutics targeting *E2F*-*p73* axis might be of great potential. Future studies on the effects of specific *p53* mutation/inactivation variations on the expression of different *p73* isoforms, and characterization of physical interaction between *E2F-1* and *p53* in the regulation of *p73* transcription will advance our understanding of *p53*–*p73* communication in tumor suppression and therapeutic responses.
